# A Murine Skin Infection Model Capable of Differentiating the Dermatopathology of Community-Associated MRSA Strain USA300 from Other MRSA Strains

**DOI:** 10.3390/microorganisms9020287

**Published:** 2021-01-30

**Authors:** Jack Zhang, John Conly, JoAnn McClure, Kaiyu Wu, Bjӧrn Petri, Duane Barber, Sameer Elsayed, Glen Armstrong, Kunyan Zhang

**Affiliations:** 1 Department of Pathology & Laboratory Medicine, University of Calgary, Calgary, AB T2N4N1, Canada; jack20110915@gmail.com (J.Z.); john.conly@albertahealthservices.ca (J.C.); jammcclu@ucalgary.ca (J.M.); kwu@ucalgary.ca (K.W.); dfbarber1@gmail.com (D.B.); 2 Department of Microbiology, Immunology and Infectious Diseases, University of Calgary, Calgary, AB T2N4N1, Canada; bpetri@ucalgary.ca (B.P.); sameer.elsayed@lhsc.on.ca (S.E.); armstrog@ucalgary.ca (G.A.); 3 Department of Medicine, University of Calgary, Calgary, AB T2N4N1, Canada; 4 Centre for Antimicrobial Resistance, Alberta Health Services, Alberta Precision Laboratories, University of Calgary, Calgary, AB T2N4N1, Canada; 5 The Calvin, Phoebe and Joan Snyder Institute for Chronic Diseases, University of Calgary, Calgary, AB T2N4N1, Canada; 6 Department of Medicine, University of Western Ontario, London, ON N6A5C1, Canada

**Keywords:** methicillin-resistant *Staphylococcus aureus* (MRSA), community-associated MRSA (CA-MRSA), CA-MRSA strain USA300, murine skin infection model, dermatopathology, dermonecrosis, neutrophil, host antibacterial response, cytokine, chemokine

## Abstract

USA300 is a predominant and highly virulent community-associated methicillin-resistant *Staphylococcus aureus* (CA-MRSA) strain that is a leading cause of skin and soft tissue infections. We established a murine intradermal infection model capable of demonstrating dermatopathological differences between USA300 and other MRSA strains. In this model, USA300 induced dermonecrosis, uniformly presenting as extensive open lesions with a histologically documented profound inflammatory cell infiltrate extending below the subcutis. In contrast, USA400 and a colonizing control strain M92 caused only localized non-ulcerated skin infections associated with a mild focal inflammatory infiltrate. It was also determined that the dermonecrosis induced by USA300 was associated with significantly increased neutrophil recruitment, inhibition of an antibacterial response, and increased production of cytokines/chemokines associated with disease severity. These results suggest that induction of severe skin lesions by USA300 is related to over-activation of neutrophils, inhibition of host antibacterial responses, and selective alteration of host cytokine/chemokine profiles.

## 1. Introduction

Methicillin-resistant *Staphylococcus aureus* (MRSA) is a leading cause of infection worldwide. It was traditionally considered a nosocomial pathogen in healthcare facilities (known as hospital-associated MRSA (HA-MRSA)). However, community-associated MRSA (CA-MRSA) emerged in the 1990s, and quickly increased and then replaced HA-MRSA in the United States and Canada. Prior to 2000, the predominant CA-MRSA strain in North America was pulsotype USA400 (ST1-MRSA-IV). It was quickly replaced by pulsotype USA300 (ST8-MRSA-IV), which is the strain responsible for the increase in CA-MRSA infections in North America [1,2], as well as globally [3,4,5]. USA300 causes primarily acute bacterial skin and skin structure infections (ABSSSIs) [6], with an estimated 44.6% of ABSSSIs in North America caused by *S. aureus* [7], and the majority of those caused by USA300 [8]. Additionally, infections induced by USA300 are believed to be more invasive and more severe when compared with those induced by other MRSA strains [9,10,11,12,13,14].

Several skin and soft tissue animal infection models have been developed to study CA-MRSA virulence, such as the murine surgical wound infection model [15], mouse foreign body infection model [16], mouse muscle infection model with microspheres as an *S. aureus* carrier [17], mouse subcutaneous (skin or ear) infection model [18,19], and mouse burn-injured infection model [20]. However, most of these animal models were designed to test the role of individual *S. aureus* virulence factors, or the effect of antimicrobials, rather than for comparing virulence between strains. In fact, there are limited studies comparing the pathogenic propensity of CA-MRSA strains responsible for serious skin and soft tissue infections, with no mouse infection model capable of differentiating USA300 virulence from that of other CA-MRSA strains. Building on previously described models, we established a mouse skin infection model capable of differentiating the virulence of USA300 from other CA-MRSA strains (USA400) as well as from a colonization strain (M92), which simulates human skin and soft tissue infection characteristics. Furthermore, we compared the immune response induced by the various strains to explore the potential mechanisms contributing to USA300 hypervirulence.

## 2. Materials and Methods

### 2.1. MRSA Strains and Their Phenotypic and Genotypic Characterization

USA300-C2406 was isolated from a patient with a lethal case of necrotizing pneumonia during our local CA-MRSA strain USA300 outbreak in Calgary in 2004 [21]. Control strain USA400-CMRSA7 was provided by the National Microbiology Laboratory, Health Canada, Winnipeg, Canada [22]. Control strain M92 was found as a nasal colonizer of staff in our local hospitals, but was never associated with invasive infection. Phenotypic and genotypic characterization of the isolates was done as previously described [23]. Whole genome sequencing was performed for the isolates using Pacific Biosciences (PacBio, Menlo Park, CA, USA) RSII sequencing technology, at the McGill University Genome Quebec Innovation Center. Genome assembly was accomplished using the hierarchical genome assembly process (HGAP v. 2.3.0.140936.p5), with the complete genome of C2406 (GenBank No.: PRJNA345240; CP019590.1), CMRSA7 (GenBank No.: PRJNA362898), and M92 (GenBank No.: PRJNA319679; CP015447.1) submitted to GenBank and annotated using the prokaryotic genome annotation pipeline [24,25]. Virulence gene profiles for the isolates, including 17 toxin genes, 12 adhesin genes, and 4 exoenzyme genes, were identified and analyzed through the strain whole genome sequences.

### 2.2. Murine Skin Infection Model

Single colonies of each bacterial strain were grown overnight in brain–heart infusion (BHI) broth at 37 °C, followed by subculture in 50 mL BHI at 37 °C until the optical density at 600 nm (OD600) was 0.7. Bacteria were centrifuged and the pellet washed twice with saline, then resuspended in 0.5 volume of saline (roughly 2 × 10^8^ CFU/mL). Animal infection experiments were performed at the Animal Resource Centre at the University of Calgary, in accordance with institutional and national guidelines of the Canadian Council on Animal Care, under protocol numbers M06074, M09115, and AC13-0076 (approved by the Animal Care Committee, University of Calgary). The experiments were repeated 3 times. Female BALB/c mice (Charles River Laboratories, Inc., Wilmington, MA, USA), aged 6 to 8 weeks, were shaved in the intrascapular region with electrical clippers prior to injection. Fifteen mice were assigned to each of the 4 groups and injected intradermally with 1 × 10^7^ CFU/50 µL of the appropriate strain or mock-infected control (saline control), in the center of the shaved area. Mice were carefully monitored for skin (wound) infection and signs of distress, and euthanized on days 4, 7, and 15~17. The skin lesion areas (mm^2^) were estimated by multiplying the width (mm) and length (mm) of the lesion. For each mouse, the whole spleen was aseptically harvested and homogenized in 1 ml sterile saline. Quantitative cultures [serial dilutions and spread on Tryptic soy agar (TSA)plates] were performed to determine bacterial load, as splenic bacterial load was used as an indicator of systemic MRSA dissemination. Full thickness biopsies of the core cutaneous lesions were performed, fixed in 10% neutral buffered formalin, and subjected to detailed histopathological examination.

### 2.3. Histology

The lesional skin tissue was cut into tissue blocks by the Airway Inflammation Research Group (AIRG) Histology Services at the University of Calgary. Tissue sections 4 µm thick were affixed to microscope slides and deparaffinized. The sections were stained with hematoxylin and eosin (H&E), Gram, and chloracetate esterase staining for routine histology, to identify bacteria, and to highlight neutrophils, respectively. For the H&E staining (NovaUltra^TM^ H&E Stain Kit, Rockville, MD, USA), fixed slides were deparaffinized by 2 changes of xylene for 5 min, and re-hydrated in 2 changes of 100% alcohol for 5 min each, then 95% and 70% alcohol for 1 min each. The slides were stained with Mayer’s hematoxylin solution for 2 min, washed with running tap water for 2 min, and rinsed in 95% alcohol. The slides were further counterstained with eosin solution for 45 s, and rinsed in 95% alcohol. The stained slides were dehydrated through 2 changes of 100% alcohol, 5 min each, and cleared with 2 changes of xylene, 5 min each. Finally, the slides were mounted with xylene-based mounting medium. For the Gram staining (BD Gram Stain Kits, Franklin Lakes, NJ, USA), fixed slides were deparaffinized and re-hydrated as described above. The slides were stained with Gram crystal violet for 1 min, and flooded with Gram iodine for 1 min. The slides were decolorized by Gram decolorizer, then counterstained with Gram safranin for 1 min. For the chloracetate esterase staining, deparaffinized sections were stained with the freshly prepared staining solution (5.0 mg naphthol AS-D chloracetate, 5 mL N-N dimethylformamide (NNDMF), 6 drops 4.0% sodium nitrite, 6 drops 4% new fuchsin in 47.5 mL phosphate buffer) for 25 min, followed by counterstaining with hematoxylin nuclear stain. The stained slides were dehydrated and cleared as described above.

### 2.4. Myeloperoxidase (MPO) Assay

The tissue myeloperoxidase activity assay was performed as an index of neutrophil recruitment, as previously described [26]. The test was repeated twice, with at least 5 mice from each group and each time point included from each experiment. Briefly, the skin was cut and weighed prior to homogenization in a 0.5% hexadecyltrimethylammonium bromide phosphate-buffered (pH 6.0) solution using a polytron PT1300D homogenizer (Kinematica, Lucerne, Switzerland). The homogenates were centrifuged at 14,000 rpm for 5 min at 4 °C in a microcentrifuge and five aliquots of each supernatant were transferred into 96-well plates, followed by the addition of a 3, 3′-dimethoxybenzidine and 1% hydrogen peroxide solution. Standard dilutions of pure myeloperoxidase were also tested for their activity to construct a standard curve (OD as a function of units of enzyme activity). Optical density readings at 450 nm were taken at 1 min (which corresponds to the linear portion of the enzymatic reaction) using a Spectra Max Plus plate reader using SOFTmax Pro v. 3.0 software (Molecular Devices Corp., Sunnyvale, CA, USA). Myeloperoxidase activity was expressed as units of enzyme per gram of tissue.

### 2.5. Spinning Disk Confocal Microscopy

An amount of 10^7^/CFU of bacteria were injected intradermally into the lateral abdominal skin, to avoid forming lesions on the mid-line. Examination of the microcirculation of the lateral abdominal skin was prepared for microscopy in order to determine the recruitment of immune cells in lesions, as described [27]. Flank skin microvasculature was visualized using a spinning disk confocal microscope, using an Olympus BX51 upright microscope with an x20/0.95 XLUM Plan Fl water immersion objective. The microscope was equipped with a confocal light path (WaveFx; Quorum, ON, Canada) based on a modified Yokogawa CSU-10 head (Yokogawa Electric Corporation, Tokyo, Japan). Anti-CD4-FITC (L3T4; BD Biosciences; 3 µg/mouse) and anti-mouse Gr-1 efluo660 (RB6-8C5; eBioscience, CA, USA; 2 µg/mouse) and anti-mouse CD31 coupled to Alexa647 (390; BD Biosciences conjugated via an Invitrogen protein labeling kit: 10 µg/mouse) were injected intravenously (IV) into BALB/c mice to image CD4+ T lymphocytes, neutrophils, and endothelial cells, respectively. Both 491 and 640 nm laser excitation wavelengths (Cobalt, Stockholm, Sweden) were used in rapid succession and visualized with the appropriate long-pass filters (520 +/− 35 nm and 640 +/− 40 nm, respectively, Semrock, IL, USA). Typical exposure time for both excitation wavelengths was 900 ms. A 512 × 512 pixel back-thinned electron-multiplying charge-coupled device camera (C9100-13, Hamamatsu, Japan) was used for fluorescence detection, with Volocity Acquisition software (Improvision, MA, USA) used to drive the confocal microscope. CD4 T lymphocyte and neutrophil rolling and adhesion were simultaneously assessed in 5–10 random fields of view in the postcapillary venules of the skin. The adherent cells were defined as cells which remained in the same location for 30 s in 100 µm of the venule, while the emigrated cells were defined as the cells which stayed outside of the vasculature in the field of view (FOV).

### 2.6. In Vivo Neutrophil Depletion

To determine the role of neutrophils in USA300-C2406 infection, a depletion process was employed using the antibody RB6-8C5 (BioXCell, West Lebanon, NH, USA), which mainly depletes neutrophils, at different time points in mice with a USA300-C2406. There were at least 3 mice for each group and each time point. As noted above, mice were injected with 10^7^ CFU of USA300-C2406 intradermally in the intrascapular region on day 0. Mice in the early depletion groups received an intraperitoneal (i.p.) injection of 200 µg RB6-8C5 24 h before infection, followed by injections every 48~72 h. Mice in the late depletion groups received an i.p. injection of 200 µg RB6-8C5 24 h after infection, followed by injections every 48~72 h. Mice in the control group were injected i.p. with 200 µg of rat IgG2 isotype control monoclonal antibody (BioXCell), on the same schedule with the depletion antibody RB6-8C5. All mice were carefully monitored (including obtaining weights), then euthanized on days 4, 7, and 14 post-infection. Lung, liver, and spleen were harvested and homogenized in 1 mL sterile saline, then quantitative cultures (serial dilutions and spread on TSA plates) were performed to determine bacterial load, as described above. The skin samples were processed into tissue sections and affixed to microscope slides for Gram staining, as described above.

### 2.7. Mouse Antibacterial Response PCR Array

To assess whether the bacterial strains could inhibit host immune responses, the transcriptional levels of 84 host genes related to the antibacterial response were determined in local skin using a Qiagen mouse antibacterial response PCR array (Qiagen Inc., Germantown, MD, USA). Included were genes involved in Toll-like receptor (TLR) signaling, NOD-like receptor (NLR) signaling, apoptosis, inflammatory response, cytokines/chemokines, and antimicrobial peptides. Samples from at least 3 mice in each group on day 4 were assessed. RNA from lesion samples was isolated using the RNeasy Plus Mini Kit (Qiagen Inc.). Ten milligrams of each sample were homogenized in RLT buffer. The contaminating DNA was removed with gDNA eliminator spin columns. RNA was purified by washing with RW1 buffer, and collected with the RNeasy spin column. RNA was further purified and reverse transcribed to cDNA using the RT2 First Strand Kit (Qiagen Inc.). Eight microliters of raw RNA were mixed with 2 µL buffer GE and incubated for 5 min at 42 °C to eliminate contaminating DNA. Four microliters of buffer BC3, 1 µL control P2, 2 µL RE3 reverse transcriptase mix, and 3 µL RNase-free water were added and the reaction was incubated at 42 °C for 15 min. The reaction was stopped by incubation at 95 °C for 5 min. The expression of 84 host genes in the mouse antibacterial response PCR array (Qiagen Inc.) was assessed by qRT-PCR using RT2 SYBR Green qPCR Mastermix (Qiagen Inc.) on a CFX96 Real-Time Detection System (Bio-Rad, Hercules, CA, USA). Thermal cycle conditions were performed as described for the RT2 Profiler PCR Array (Qiagen Inc.). One hundred and two microliters of the cDNA synthesis reaction were mixed with 1350 µL 2× RT2 SYBR green mastermix and 1248 µL RNase-free water. Twenty-five microliters were then aliquoted into each well of the RT2 Profiler PCR Array. The PCR plates were heated to 95 °C for 15 min, followed by 40 cycles of denaturation–extension (95 °C for 15 s and 60 °C for 1 min). Because the transcription of housekeeping genes during wound forming and healing varies [28], expression of the 84 genes was normalized to the mean of the 5 most stably expressing housekeeping genes (*actb*, *b2m*, *gapdh*, *gusb*, and *hsp90ab1*). The PCR array data were further uploaded to the Qiagen RT2 Profiler PCR Array Data Analysis Center (http://www.qiagen.com/geneglobe. Samples). Ct = 35 was set as the cut-off Ct value, while 5 housekeeping genes were selected as normalization factors. Relative target gene expression was calculated according to the ∆∆Ct method [29], in which the fold difference in expression was 2^-∆∆Ct^.

### 2.8. Cytokine and Chemokine Assay

The production of various cytokines and chemokines in local skin samples was determined using a Luminex 32-plex by Eve Technologies (University of Calgary, AB, Canada). The 32-plex consisted of eotaxin, G-CSF, GM-CSF, IFN-gamma, IL-1alpha, IL-1beta, IL-2, IL-3, IL-4, IL-5, IL-6, IL-7, IL-9, IL-10, IL-12 (p40), IL-12 (p70), IL-13, IL-15, IL-17, IP-10, KC, LIF, LIX, MCP-1, M-CSF, MIG, MIP-1alpha, MIP-1beta, MIP-2, RANTES, TNF-alpha, and VEGF. Samples were from 5 mice in each group at each time point. Tissue lesions were homogenized in 20 mM Tris HCl (pH 7.5), 0.5% Tween 20, 150 mM NaCl, and Sigma protease inhibitor 1:100 buffer. The supernatant was collected and cytokines and chemokines were separated into several groups for analysis, as follows: (1) protective cytokines and chemokines whose depletion results in severe infection in a mouse skin infection model [30,31]; (2) cytokines and chemokines related to *S. aureus* disease severity, but which lack depletion experiments to confirm their role in a mouse skin infection model [32,33,34,35,36,37,38]; (3) growth factors which are important for wound healing;(4) cytokines and chemokines related to the Th1 and Th2 response; and (5) cytokines and chemokines whose roles are unclear in MRSA infection. The latter was further divided into 2 sub-groups; one sub-group generally believed to be related to tissue injury, infection, and allergic disease and the other believed to be related to immune cell development, virus infection, and aging.

### 2.9. Statistical Analysis

All analyses were performed using SPSS 20.0 and Prism v. 5. Differences in CFU, diameter of lesion, and MPO activity were evaluated using one way ANOVA. The Qiagen RT2 Profiler PCR Array Data Analysis Center was used to analyze the Qiagen PCR Array. *p* values of *p* < 0.05 were considered statistically significant.

## 3. Results

### 3.1. Genotypic and Phenotypic Characterization and Virulence Gene Profiles of MRSA Strains

**Figure 1:**
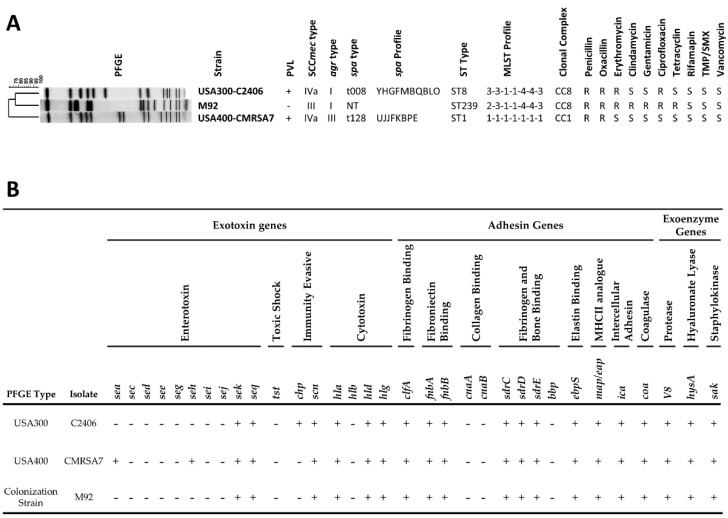
Methicillin-resistant *Staphylococcus aureus* (MRSA) strain genotypic and phenotypic characteristics, and virulence factor profiles. (**A**) Pulsed Field Gel Elecctrophoresis (PFGE) profiles for the MRSA strains, along with genotypic and phenotypic typing results. PVL, Panton–Valentine leucocidin (+, positive; -, negative); SCC*mec*, staphylococcal cassette chromosome *mec*; *agr*, accessory gene regulator; *spa*, staphylococcal protein A (non-typable, NT); MLST, multilocus sequence type; CC, clonal complex; S, susceptible; R, resistant. (**B**) Virulence gene profiles show that the isolates differ by only 3 genes (*sea*, *seh*, *chp*). *sea*/c/d/e/g/h/i/j/k/q, staphylococcal enterotoxin A/C/D/E/G/H/I/J/K/Q; *tst*, toxic shock syndrome toxin; *chp*, chemotaxis inhibitory protein; *scn*, staphylococcal complement inhibitory protein; *hla/b/d/g*, *α*/*β/*𝛿/*γ*-hemolysin; *clfA*, clumping factor; *fnbA/B*, fibronectin adhesive molecule A/B; *cnaA/B*, collagen adhesive molecule A/B; *sdrC/D/E*, putative adhesin; *bbp*, bone sialoprotein adhesin; *ebpS*, elastin adhesin; *map*, major histocompatibility complex class II analog protein; *ica*, polysaccharide intercellular adhesin. *coa*, coagulase; *V8*, serine protease; *hysA*, hyaluronidase; *sak,* staphylokinase; +, positive; -, negative.

The genotype, antibiotic resistance (Figure 1A), and virulence factor profiles (Figure 1B) of USA300-C2406 were compared to those of the control strains. Colonization strain M92 belonged to ST239-MRSA-III, carried a non-typeable *spa* gene, and was Panton–Valentine leucocidin (PVL) negative and *agr* type I. USA400-CMRSA7 belonged to pulsotype USA400 (ST1-MRSA-IVa), was PVL positive, belonged to *spa* type t128, and *agr* type III. USA300-C2406 belonged to pulsotype USA300 (ST8-MRSA-IVa), was PVL positive, belonged to spa type t008, and *agr* type I. Phenotypic tests indicated that USA400-CMRSA7 was sensitive to erythromycin and ciprofloxacin, unlike USA300-C2406, which was resistant to both. Likewise, M92 was resistant to clindamycin, gentamicin, and tetracycline, while USA300-C2406 was sensitive to these agents. Virulence factors were also assessed and compared between the strains. They were similar, with the differences being that USA300-C2406 carried the *chp* gene and CMRSA7 carried the *sea* and *seh* genes.

### 3.2. USA300 Induced Extensive Open Lesions (Dermonecrosis) while M92 and USA400 Caused Localized Infection

**Figure 2:**
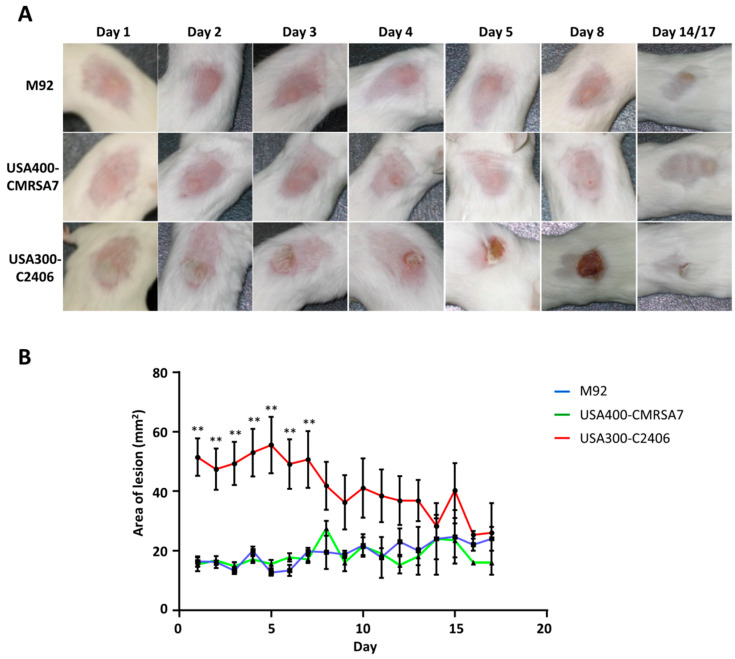
Disease progression in acute intradermal infection model showing localized infections caused by M92 and USA400-CMRSA7, and necrosis caused by USA300-C2406. (**A**) Representative photos for select time points (day 1, 2, 3, 4, 5, 8, 14, or 17) for each of the MRSA strains (M92, USA400-CMRSA7, USA300-C2406) showed that M92 and USA400 caused localized infections, while USA300 caused ulceration and necrosis (open wound). (**B**) The lesion diameter for each of the 3 strains. The average lesion area (mm^2^) for M92 (blue), USA400-CMRSA7 (green), and USA300-C2406 (red) at each time point was expressed as mean + SEM. The experiment was repeated twice, with 5 mice in each time point and each group. Not significant between USA400-CMRSA7 and M92. Statistical comparisons between USA300-C2406 and M92 or USA400-CMRSA7 were indicated with asterisk: ** *p* < 0.01.

Our mouse skin infection model differentiated USA300-C2406 hypervirulence from the control strains in that it demonstrated more severe skin lesions than M92 and USA400-CMRSA7 (Figure 2). Intradermal inoculation in either M92 or USA400-CMRSA7 resulted in confined dermal abscesses with average areas of 15.5 mm^2^ and 16.36 mm^2^, respectively. The sizes of these abscesses remained relatively unchanged from day 1 to day 7. Healing began after day 7, with full recovery on day 14~17. In contrast, USA300-C2406 caused abscesses with an average area of 51.40 mm^2^ (*p* < 0.01 compared with M92 and USA400-CMRSA7) on day 1. This was followed by the development of an ulcerated open wound with underlying necrosis that reached its maximum size on day 5. The size of the lesion did not change significantly from day 4 to day 7. The wound started to heal after day 7, with recovery on day 14~17. Cultures of the spleen demonstrated that there was no systemic infection in this model.

### 3.3. Profound Inflammatory Cell Infiltration by USA300

**Figure 3:**
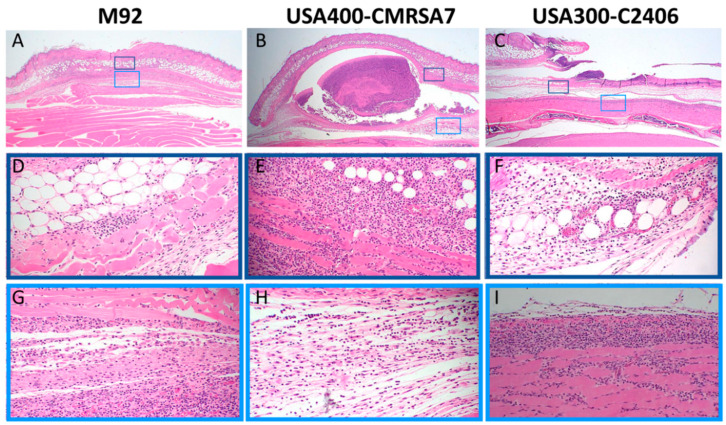
Histopathology (hematoxylin and eosin (H&E) stain) of the core lesions, induced on day 4, showing more severe inflammatory changes secondary to infection with USA300-C2406. The overall lesions (10×) induced by M92, USA400-CMRSA7, and USA300-C2406 are presented in panels (**A**–**C**, respectively. The 40X amplifications of select regions are presented in panels **D**–**I**. M92 induced minor inflammatory cell infiltration in adipose layer, muscular layer, and fascia (**D**,**G**). USA400-CMRSA7 induced abscess formation in the fascia, surrounded by inflammatory cell infiltration (**E**,**H**). In contrast, USA300-C2406 induced ulceration, more tissue necrosis, and a denser, more deep-seated inflammatory cell infiltrate that extended into the skeletal muscle (**F**,**I**).

The histological changes induced by various MRSA strains were assessed on day 4 since this was the point when the skin lesions reached their maximum diameter/area. The severe tissue damage induced by USA300-C2406 was associated with more disseminated inflammation when compared with that caused by M92 and USA400-CMRSA7. As shown in the Figure 3, M92-infected skin was associated with mild changes in the epidermis and dermis (panel A). The inflammatory cell infiltration extended into the adipose tissue, superficial muscle, and fascia (D and G). USA400-CMRSA7 infection formed a well-circumscribed area of skin necrosis (B), which was associated with a mild inflammatory cell infiltration in the surrounding adipose tissue, superficial muscle, and fascia (E and H). In contrast, USA300-C2406 formed extensive lesions with ulceration (C) and dense neutrophil-rich infiltrates that extended into the dermis, adipose tissue, fascia, and skeletal muscle (F and I). The necrotic debris was located at the edge and bottom (fascial level) of the ulcer and the disseminated inflammatory cells infiltrated not only the skin but also the skeletal muscle beneath the fascial plane.

### 3.4. Primary Infiltrating Inflammatory Cell Type Was the Neutrophil

**Figure 4:**
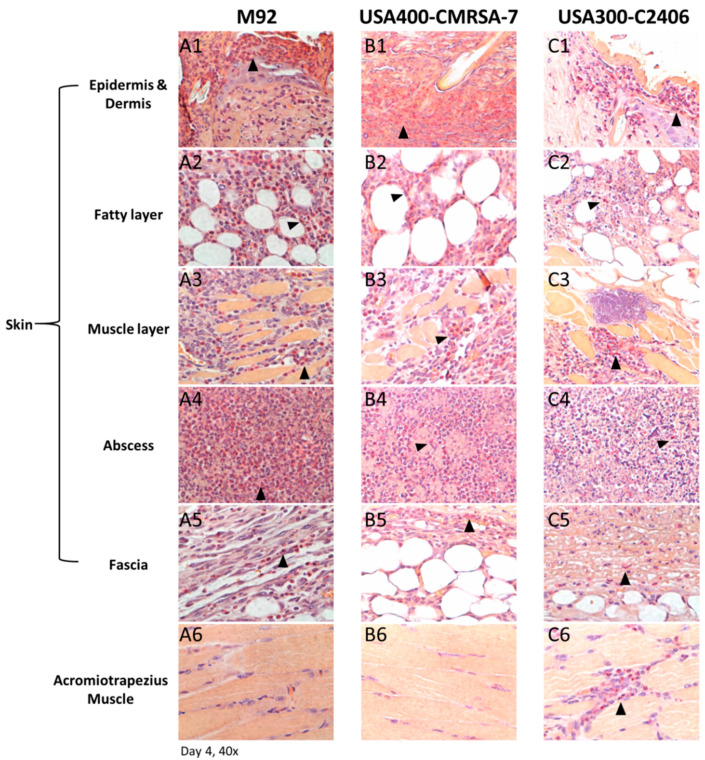
Histopathology (esterase staining) of skin lesions from day 4. Representative photos (40×) from different layers of lesions induced by M92 (A1–A6), USA400-CMRSA7 (B1–B6), and USA300-C2406 (C1–C6). Positive cells (primarily neutrophils) were located at the center of the abscess (A4, B4, and C4) and edges of lesions (A1–A3, B1–B3, and C1–C3). Positive cells are labeled with arrows. The skin lesion induced by USA300-C2406 presented a denser and deeper neutrophil infiltrate than did that of USA400-CMRSA7 and M92.

An esterase stain was done on samples collected on day 4 (Figure 4). This confirmed the histological findings of a neutrophil-rich inflammatory infiltrate in the case of USA300-C2406 that extended deeper than was the case with M92 and USA400-CMRSA7. In the former, a dense neutrophilic infiltrate was located not only in the skin but also in the deep skeletal muscle.

### 3.5. USA300 Induced Prolonged Periods of Excessive Neutrophil Infiltration

**Figure 5:**
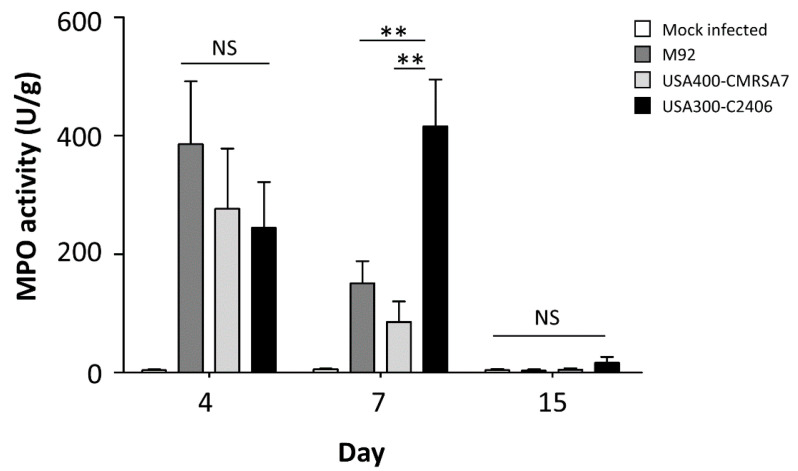
USA300 infection leads to significantly more myeloperoxidase (MPO) activity in skin samples on day 7. The myeloperoxidase activity (U/g) in local skin samples from mock-infected control (white bars), M92- (dark gray bars), USA400-CMRSA7- (light gray bars), and USA300-C2406-infected (black bars) mice at different time points (days 4, 7, and 15) is shown as mean + SEM. The experiment was repeated twice, with 5 mice in each time point and each group. NS, not significant; ** *p* < 0.01.

An MPO assay was employed to quantify neutrophil infiltration in local skin samples (Figure 5). All three MRSA strains induced higher levels of MPO activity relative to the mock-infected control. On day 4, M92, USA400-CMRSA7, and USA300-C2406 induced 386.15 U/g, 276.75 U/g, and 244.85 U/g, respectively (*p* > 0.05). On day 7, however, the MPO activity of M92 and USA400-CMRSA7 was 150.88 U/g and 85.97 U/g, respectively, while the MPO activity of USA300-C2406 reached 415.61 U/g (*p* = 0.007 and 0.001 compared with M92 and CMRSA7, respectively). On day 17, the MPO level of M92, USA400-CMRSA7, and USA300-C2406 decreased to 3.87 U/g, 5.00 U/g, and 16.96 U/g, respectively, with no significant differences noted (*p* > 0.05).

### 3.6. Significantly Greater Neutrophil (But Not CD4 T Cell) Adhesion and Emigration Triggered by USA300

**Figure 6:**
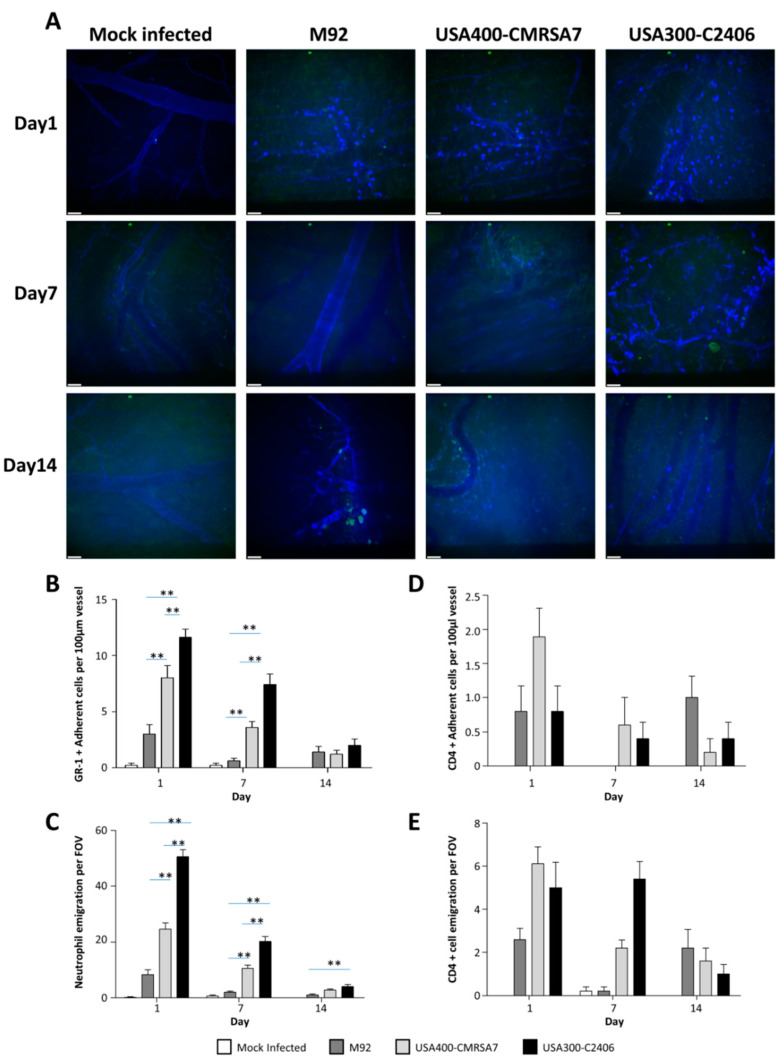
Confocal microscopy revealed that USA300 induced greater neutrophil (but not CD4 cell) adherence and emigration. (**A**) Representative examples for visualizing GR-1+ cells, CD4 + cells, and endothelial cells in mouse flank skin with spinning disc confocal microscopy on days 1, 7, and 14. Gr-1+ efluo660 neutrophils are seen as bright blue ovals whereas endothelial cells stained with CD31-A647 can be seen as thin blue lines outlining the vessel walls. CD4+ cells are depicted in FITC (green). (**B**–**E**) Bar graphs showing quantification (mean + SEM) of adherent and emigrated neutrophils (**B** and **C**) and CD4+ T cells (**D** and **E**) on days 1, 7, and 14 for the mock-infected control (white bars), M92 (dark gray bars), USA400-CMRSA7 (light gray bars), and USA300-C2406 (black bars). USA300-C2406 induced significantly greater neutrophil adherence and emigration when compared with USA400-CMRSA7 and M92 on day 1 and 7 (*p* < 0.01). Experiments with 5 mice in each group and each time point. ** *p* < 0.01. Scale bar represents 40 µm.

Neutrophil adherence and emigration were assessed using spinning disk confocal microscopy (Figure 6). Infected mice had elevated numbers of adherent and emigrated neutrophils as compared to mock-infected controls, with greater neutrophil adhesion and emigration induced by USA300-C2406 in comparison to what was seen in the other two strains. On day 1, M92 and USA400-CMRSA7 induced 3.00 cells/100 µm and 8.00 cells/100 µm, respectively, while USA300-C2406 induced 11.60 cells/100 µm (*p* = 0.055 and 0.000 compared with USA400-CMRSA7 and M92, respectively). On day 7, the number of neutrophils induced by all three strains was reduced; M92 and USA400-CMRSA7 induced 0.60 cells/100 µm and 3.60 cells/100 µm, respectively, and USA300-C2406 induced 7.40 cells/100 µm, which was significantly greater than M92 and USA400-CMRSA7 (*p* = 0.000 and 0.001, respectively). On day 14, there was no significant difference among the three strains. In terms of neutrophil emigration, on day 1, M92 and USA400-CMRSA7 induced 8.40 cells/field and 24.56 cells/field, respectively, while USA300-C2406 induced 50.60 cells/field (*p* = 0.000 compared with both M92 and CMRSA7). On day 7, M92 and USA400-CMRSA7 induced 2.00 cells/field and 10.60 cells/field, respectively, while USA300-C2406 induced 20.20 cells/field (*p* = 0.000 compared with both M92 and USA400-CMRSA7). On day 14, there was no significance among the three strains. No significant trend was observed for CD4+ T cell adhesion and emigration among the three strains.

### 3.7. Co-localization of Higher Bacterial Load with Neutrophil Infiltration in USA300 Infection

**Figure 7:**
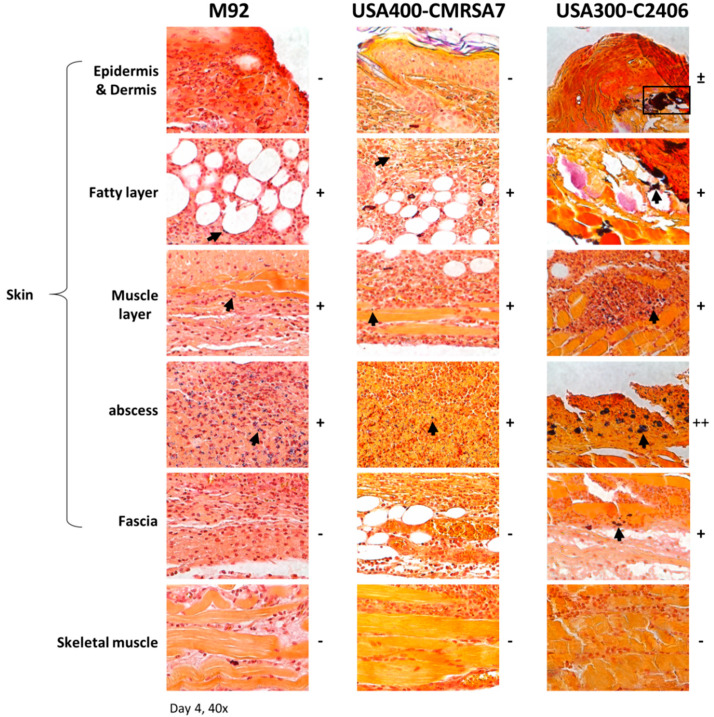
Histopathology (Gram staining) of skin lesions shows that USA300 infections have a higher bacterial load. Representative images (40× magnification) from various layers of skin and subcuticular tissue in the lesion from M92, USA400-CMRSA7, and USA300-C2406 infections on day 4 are presented. USA300-C2406 infections have a higher bacterial load as compared to USA400-CMRSA7 and M92 infections. Positive spots (bacteria) are labeled with a black box or arrow. The relative quantity of positive spots is indicated with + (positive), ++, and – (negative for bacteria).

To determine if neutrophils were targeting bacteria, tissue slides (from day 4 lesions) were stained with Gram stain to determine bacterial localization (Figure 7). Deeper bacterial dissemination in USA300-C2406-infected tissue was noted, with bacteria found throughout the skin tissue and invading the skeletal muscle. In contrast, bacteria were predominantly located in the abscess of lesions caused by M92 and USA400-CMRSA7. There was a direct correlation between the presence of bacteria and the presence of neutrophils.

### 3.8. Neutrophil Depletion Resulted in a More Severe Infection

**Figure 8:**
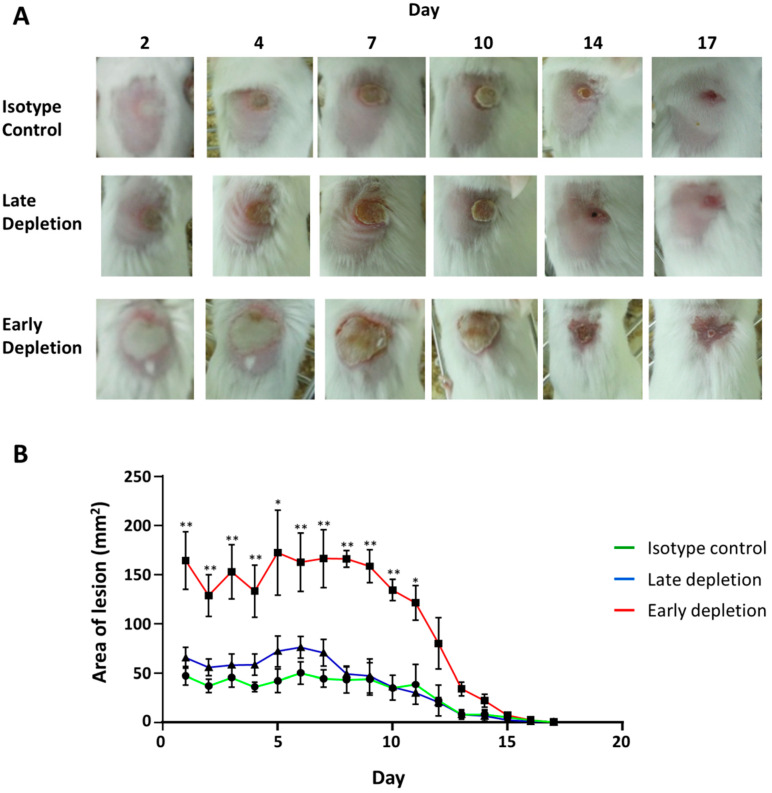
Early neutrophil depletion results in more severe lesions in mice infected with USA300. (**A**) Representative photos from days 2, 4, 7, 10, 14, and 17 showing the lesion in isotype control, late depletion, and early depletion groups infected with USA300-C2406. (**B**) Lesion area, expressed as mean area of skin lesion (mm^2^) + SEM, for the isotype control (green), late depletion (blue), and early depletion (red) groups. Experiments with 9 mice in each group. * *p* < 0.05; ** *p* < 0.01.

We depleted the neutrophils in mice infected with USA300-C2406 to examine the role they played in disease progression. We observed that early neutrophil depletion (24 h before infection) resulted in a significantly more severe infection as compared to the isotype control (Figure 8). With the isotype control group, the wound formed on day 1 and remained constant with an average area of 42.53 mm^2^ until day 10, at which time it gradually decreased and was healed around day 17. Similarly, with late neutrophil depletion (24 h after infection), the average wound area on day 1 was 65.67 mm^2^, which was not statistically different from the isotype control group (*p* = 0.216). The wound maintained an average area of 50 mm^2^ until day 8, at which time it decreased, similar to the isotype control. When neutrophils were depleted prior to infection, however, the mice showed an enlarged wound with an average area of 164.37 mm^2^ on day 1 (*p* = 0.001 and = 0.005, compared with isotype control and late depletion group, respectively), which remained constant until day 11, then gradually reduced in size and healed by day 17 (Figure 8 B).

### 3.9. Neutrophil Depletion Resulted in Bacterial Dissemination and Invasive Infection

**Figure 9:**
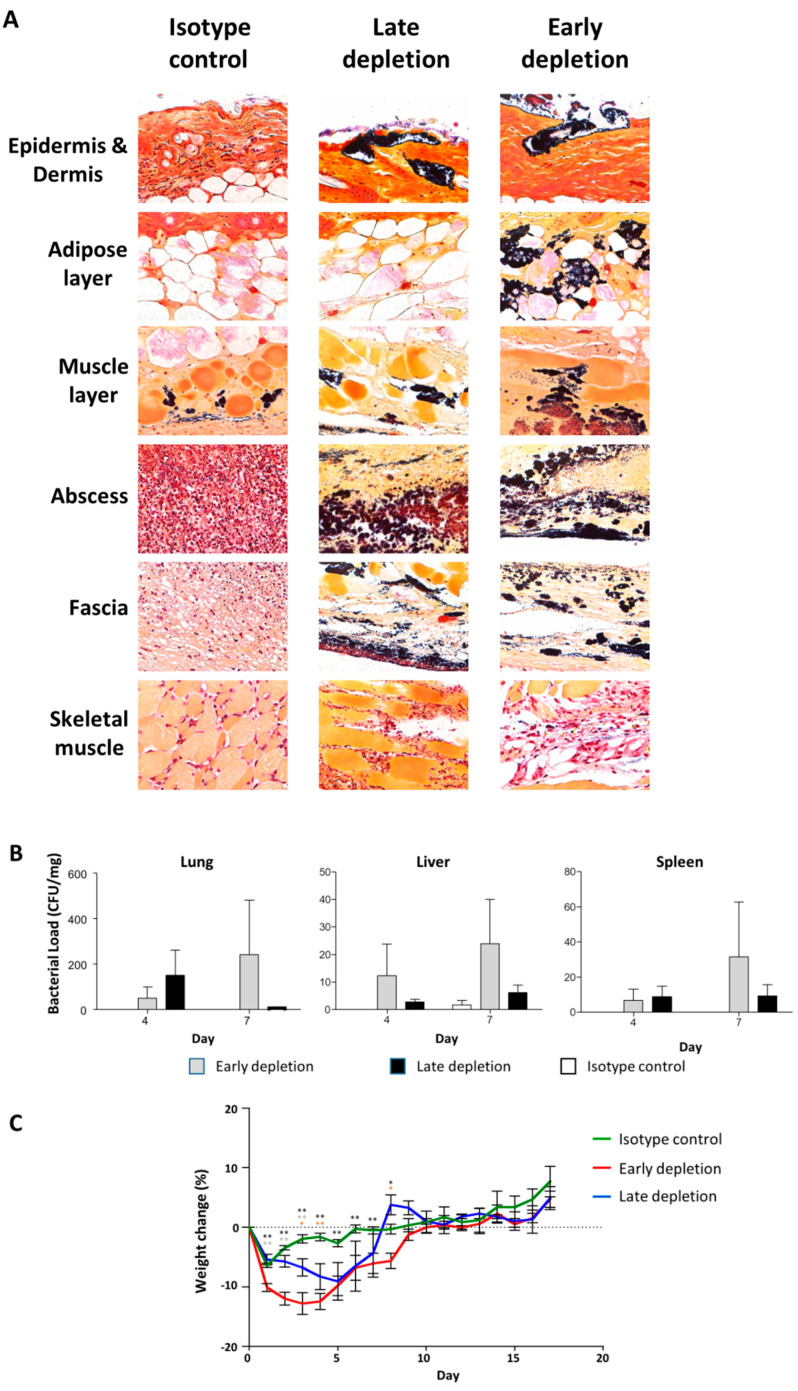
Neutrophil depletion resulted in bacterial dissemination and weight loss. (**A**) Representative images (40×) from the skin and subcuticular tissue following early neutrophil depletion, late neutrophil depletion, and isotype control group on day 4 after infection with USA300-C2406. Gram staining indicates that there were high bacterial loads when neutrophils were depleted. (**B**) USA300-C2406 bacterial counts (CFU/mg) recovered from lung, liver, and spleen at day 4, 7, and 17 time points, in early neutrophil depletion (gray bars), late neutrophil depletion (black bars), and isotype control (white bars) groups. (**C**) Percentage of weight change following infection with USA300-C2406 in the isotype (green), early neutrophil depletion (red), and late neutrophil depletion groups (blue), showing that severe weight loss was associated with neutrophil depletion. Weight changes were expressed as mean + SEM. Experiments with 9 mice in each group. Statistical comparisons between mock infection and early depletion (black asterisk), mock infection and late depletion (orange asterisk), early and late depletion (grey asterisk); *p < 0.05; **p < 0.01.

To investigate the cause of increased tissue damage following neutrophil depletion, we used Gram staining to identify bacteria in local skin (Figure 9A), and organ culture to identify bacterial load in the internal organs (Figure 9B). Gram stains indicated that there were more bacteria present in the tissue in both early and late neutrophil depletion groups as compared to the isotype control group, and that the bacteria invaded deeply into skeletal muscle in depletion groups, but were limited to the skin in the isotype control group. Organ cultures demonstrated that the bacterial load in the lung, liver, and spleen was less than 1 CFU/mg in the control group, whereas the bacterial load from the early and late depletion groups ranged from 5–100 CFU/mg. Weight change during infection was used as a marker for infection severity (Figure 9C), and showed that the late depletion group had significantly more weight loss from days 3 to 4, while the early depletion group had significantly more weight loss from days 1 to 8.

### 3.10. Unique Pattern of Molecular Transcription Results from Infection with USA300

Because USA300 infections were associated with extensive neutrophil infiltration and increased bacterial load, we hypothesized that USA300 might employ a mechanism designed to modify or inhibit the antibacterial response in the mice. Using an antibacterial response PCR array, we examined the expression of factors such as Toll-like receptor signaling, NOD-like receptor (NLR) signaling, bacterial pattern recognition receptors (PRRs), signaling downstream of antibacterial responses, apoptosis, inflammatory responses, cytokines/chemokines, and antimicrobial peptides in local skin samples. Our results demonstrated that MRSA USA300 infection could activate the transcription of most of the components involved in the antibacterial responses. However, when compared with USA400-CMRSA7 and M92, USA300-C2406 induced lower degrees of upregulation.

For components related to TLR signaling, USA300-C2406 induced less transcription of *akt1*, *irak3*, and *tlr9* (2.03–4.49-fold) when compared with both USA400-CMRSA7 and M92, less transcription of *tlr6* and *tollip* (*p* < 0.05) (2.69–2.74-fold) when compared with USA400-CMRSA7, and less transcription of *fadd* (2.07-fold) when compared with M92. Only the transcription of LPS binding protein (*lbp*) was higher in USA300-C2406 infections than in USA400-CMRSA7 and M92 infections (*p* < 0.05) (3.31-fold and 2.26-fold, respectively).

For components related to NLR signaling, USA300-C2406 induced less transcription of *card6*, *naip1*, *nlrp1a*, and *nod2* (2.0–6.41-fold) when compared with USA400-CMRSA7, and less transcription of *casp1*, *pycard*, and *ripk2* (*p* < 0.01) (2.06–2.92-fold) when compared with M92.

For components related to signaling downstream of antibacterial responses, USA300-C2406 induced less transcription of *map2k3* (*p* < 0.05), *mapk3* (*p* < 0.05), and *nfkbia* (2.08–2.13-fold) when compared with USA400-CMRSA7.

For components related to apoptosis, USA300-C2406 induced less transcription of *akt1*, *ifnb1*, *il12b* (2.07–5.83-fold) when compared with both USA400-CMRSA7 and M92. Furthermore, USA300-C2406 induced less transcription of *card6*, *il12a*, and *nfkbia* (2.01–3.2-fold) when compared with USA400-CMRSA7 and less transcription of *casp1*, *fadd*, *il6*, *pycard*, and *ripk2* (2.05–2.92-fold) when compared with M92.

For components related to inflammatory response, USA300-C2406 induced less transcription of *akt1* and *tlr9* (2.07–4.49-fold) when compared with USA400-CMRSA7 and M92, less transcription of *tlr6* and *tollip* (2.69–2.74-fold) when compared with USA400-CMRSA7, and less transcription of *ccl5*, *cxcl1*, *il6*, and *ripk2* when compared with M92. Only the transcription of *cxcl1* and *lbp* was higher (2.23–3.31-fold) in USA300-C2406 infections than in USA400-CMRSA7 and M92 infections.

For cytokines and chemokines, USA300-C2406 induced less transcription of *ifna9*, *ifnb1*, *il12b*, and *il18* (2.07–5.83-fold) when compared with USA400-CMRSA7 and M92, and less transcription of *ccl5*, *cxcl3*, and *il6* (2.05–4.39-fold) when compared with M92. Only the transcription of *cxcl1* (2.23–2.55-fold) was higher in USA300-C2406 infections compared to USA400-CMRSA7 and M92 infections.

For antimicrobial peptides, USA300-C2406 induced less transcription of *bpi* (2.07–4.49-fold) when compared with USA400-CMRSA7 and M92, and less transcription of *camp*, *ctsg*, *ltf*, and *prtn3* when compared with USA400-CMRSA7. Transcription of *slpi* was higher (2.52–3.69-fold) in USA300-C2406 infections when compared with USA400-CMRSA7 and M92 infections.

There was, however, no statistically significant difference in the transcription of most of these aforementioned genes, with the exception of *il-18*. With *il-18*, the transcription in USA400-CMRSA7 and M92 infected mice was close to that of the mock-infected control, while USA300-C2406 induced a 2.69-fold downregulation (*p* = 0.0211). The level of transcription in USA300-C2406-infected mice was downregulated 3.22- and 3.39-fold (*p* = 0.011 and 0.030) when compared with USA400-CMRSA7 and M92, respectively.

The detailed list of transcriptional levels for genes related to each response is summarized in Supplementary Table S1.

### 3.11. Unique Cytokine and Chemokine Profiles in USA300-Infected Lesions

After determining that USA300-C2406 infections were associated with unique antibacterial responses, we further compared the local cytokine and chemokine profiles (protein profile) with a Luminex assay. On day 4, most of the cytokines and chemokines (IFNy, IL-17, IL-1b, G-CSF, GM-CSF, M-CSF, KC, TNFa, MIP2, RANTES, IL-4, IL-13, IL-6, IL-12p70, IL-12p40, VEGF, MIP1a, MIP1b, IL-5, LIF, MCP1, MIG, IL-3, eotaxin, and IP10) were increased (1.50–1584.13-fold increase) in mice infected with any of the MRSA strains as compared to the mock-infected control. The details are listed in Table 1.

For cytokines/chemokines reported to be associated with protection (IL-17, IL-1a, and IL-1b), there was no difference between USA300-C2406 and M92 or USA400-CMRSA7 (*p* = 0.8809–0.9996 and *p* = 0.344–0.769, respectively). For cytokines/chemokines associated with disease severity, USA300-C2406 significantly increased production of G-CSF, M-CSF, KC, MIP2, IFNy, GM-CSF, TNFa, IL-4, and IL-6 when compared to M92 and USA400-CMRSA7 (1.99–40.0-fold, *p* = 0.000–0.027, and 3.52–20.17-fold, *p* = 0.000–0.123, respectively), but there was no difference in the production of IL-10, IL-12p70, IL-12p40, and IL-15. For VEGF, USA300-C2406 induced 2.70- and 12.23-fold increases compared to M92 and USA400-CMRSA7, respectively (*p* = 0.013 and <0.001, respectively). Cytokines and chemokines involved in tissue injury, infection, and allergic disease, such as IL-5, LIF, LIX, MCP-1, MIP-1a, and MIP-1b, were increased in the USA300-C2406-infected mice. USA300-C2406 induced 3.26–15.99-fold and 7.67–1825.60-fold increases compared with M92 and USA400-CMRSA7, respectively (*p* = 0.000–0.093, and 0.000–0.02, respectively). Cytokines and chemokines associated with immune cell development, virus infection, and aging, including IP-10, eotaxin, and IL-3, showed no difference among these strains (*p* = 0.343–0.918 and *p* = 0.089–1.000 for USA300-C2406 compared with M92 and USA400-CMRSA7). There were no differences in the other Th1 and Th2 cytokines (IL-2, IL-9, and IL-13) between infected groups and the mock-infected control group. On day 7, most cytokines and chemokines were reduced compared to day 4, but USA300-C2406 still induced more G-CSF, IL-6, and LIF than M92 and USA400-CMRSA7 (4.86–8.53-fold, *p* = 0.000–0.046, and 2.62–6.37-fold, *p* = 0.000–0.185, respectively) (Supplementary Table S2). On day 15, the overall cytokines and chemokines were reduced, and there was no difference between the strains (Supplementary Table S3).

## 4. Discussion

There are several mouse models that have been used to study *S. aureus* infections [14,15,16,17,18,19]. Most of the models, however, were either designed to study the mechanisms of *S. aureus* virulence by comparing mutants to their corresponding wild type strains, or to study more invasive infections similar to what is seen in hospital-acquired diseases. *S. aureus* USA300 is the most common pathogen responsible for skin and soft tissue infections (SSTIs), which differ from wound or surgical site infections in that they generally begin as small lesions with areas of necrosis [36,39,40]. In this study, building on previously described models, we established a mouse skin infection model that mimics the clinical presentations seen with CA-MRSA SSTIs, and which is able to differentiate between two CA-MRSA strains, USA400 and USA300, as well as the colonization control strain, M92. With this mouse intradermal infection model, M92 and USA400-CMRSA7 caused localized infections, while USA300-C2406 caused ulceration with necrosis. The model was also able to show that the severe infections induced by USA300 were associated with a specific pattern of immune response, as well as an increased bacterial burden.

USA300 induced ulcer formation on day 1, reaching a maximum size and severity on day 4, remaining constant until day 7, and then healing by day 14–17. The USA300 infection was marked and lasted longer with more pronounced inflammation and a higher bacterial burden than the other MRSA infections, suggesting that USA300 might employ a mechanism to modify/inhibit the host’s immune response. The role of neutrophils in MRSA infection is, however, complex. We showed that neutrophil depletion resulted in more severe tissue damage and invasive infections, possibly from loss of the protective role of neutrophils, resulting in uncontrolled bacterial infections. Neutrophils play an important role in the response to *S. aureus* SSTIs [41,42,43], however, extensive infiltration and activation of neutrophils at the site of infection is believed to cause chronic inflammation, impaired injury repair, and loss of organ function [44].

Transcriptional PCR array results further supported the notion that USA300 is capable of modulating the mouse immune system and neutrophil response. Il-18, which has been shown to restore neutrophil phagocytosis during severe skin damage [20], and Il-12, which is related to natural killer NK cell and macrophage defensive function in mice [45], each showed decreased transcriptional levels in the USA300-infected mice. USA300 also induced higher transcription of *slpi*, which was shown to be involved with the inhibition of neutrophil extracellular trap (NET) formation [46]. In addition, several factors involved with Toll-like and NOD-like receptor signaling showed decreased transcriptional levels in USA300-infected mice. Recognition of *S. aureus* by Toll-like and NOD-like receptors is required for the activation of protective host responses, such as the production of inflammatory cytokines and chemokines, and is important in the defense against *S. aureus*-induced SSTIs [47,48]. It was demonstrated that some staphylococcal virulence factors, such as staphylococcal TIR domain protein (TirS) that can inhibit TLR signaling via molecular mimicry [49], and staphylococcal superantigen-like protein 3 (SSL3) that can negatively interfere with TLR2 recognition and heterodimer formation [50,51], can inhibit and block TLR2-mediated inflammatory responses and impair the cytokine production, as well as neutrophil and macrophage activity, and consequently inhibit polymorphonuclear leukocyte (PMN) infiltration [52]. Taken together, these results indicate that USA300 might possess a mechanism to inhibit the defensive immune responses, especially involving neutrophils. However, the PCR array assayed the overall transcription of factors in whole skin samples, which includes various immune cells, keratinocytes, and other structural cells. This may not represent the transcriptional level for specific cell types and may explain why there was no difference in *mpo* transcription among the various strains and mock-infected control.

Cytokine and chemokine profiles in local lesions were consistent with our hypothesis that USA300 is modulating the mouse immune response. IL-17A, IL-1α, and IL-1β are believed to be protective during mouse MRSA skin infections [30,31]. The cytokines not only play a role in neutrophil recruitment, but can also promote the production of antimicrobial peptides (such as β-defensins and cathelicidins) [53]. In this mouse skin infection model, USA300 induced the same level of production for these cytokines on day 4 when compared with the other strains, yet more neutrophils were recruited, and there was a lower level of antimicrobial peptide production in USA300-infected mice. USA300 appears to be suppressing certain factors involved with the protective response. However, infection with USA300 did trigger increased production of cytokines and chemokines related to severity, including IFNy, GM-CSF, TNFa, IL-4, and IL-6. These are believed to be related to severe infections [35,36]. Other cytokines and chemokines such as IL-5 [54], LIF [55], LIX, MCP-1 [56], MIP-1a, and MIP-1b [57], which are believed to be involved in tissue damage, infection, and allergic diseases, also showed increased production in USA300 infections. Interestingly, the expression of MIG (also related to tissue damage, infection, or allergic diseases), was the only member of this group whose expression in USA300 infections was less than that in infections with the other MRSA strains. This phenomenon deserves further study. Macrophages expressing MIG (also known as CXCL9) are believed to protect against abscess formation [58] and promote tissue repair. Our USA300 resulted in lower production of MIG on day 7 and 14 when compared with other strains, which could indicate that USA300 is inhibiting MIG to increase tissue damage. We also recognize that the level of infiltration was very different between the different strains, as shown in this study. This could complicate the transcriptomic analysis as the composition of the material used for analysis was different with more or less immune cells. For example, a lack of a statistically significant difference in IL-17a, IL1a, and IL-1b should be viewed in light of the fact that there was more immune cell infiltration in the lesions for USA300 than the other strains. Further study by using tissue type-specific control genes is needed to address this issue.

## 5. Conclusions

Our results demonstrated that USA300 infections induce an intense neutrophilic response, inhibits host antibacterial responses, selectively inhibits the production of protective cytokines, and selectively activates the production of pro-inflammatory cytokines and chemokines. This unique pattern of immune responses could cause increased tissue damage at the site of the wound and increased bacterial persistence, which would facilitate the spread of USA300. This may provide some insight into why USA300 is more dominant and more virulent than other MRSA strains.

## Figures and Tables

**Table 1:** Cytokine and chemokine profiles for mice on day 4 post-infection with M92, USA400, and USA300 MRSA strains.

					USA300-C2406			
Role	Cytokine/Chemokine	Mock Infected conc.	M92 conc.	USA400- CMRSA7 conc.	USA300- C2406 conc.	Vs. Mock Infected	Vs. M92	Vs. USA400- CMRSA7
Protection	IL-17A	0.0185 (0.0017–0.0416)	1.072 † (0.0768–2.2690)	0.1850 (0.0454–0.3744)	0.8985 (0.4565–1.4555)			
	IL-1a	37.9831 (25.0558–45.3151)	29.6027 (10.8897–54.1207)	20.2297 (6.1071–33.1123)	30.2987 (20.9430–39.5406)			
	IL-1b	0.5015 (0.2672–0.8363)	9.356 † (2.06583–18.1579)	1.3075 (0.1432–2.9846)	7.3872 (3.9964–11.6633)			
Severity	IL-4	0.0129 (0.0097–0.0183)	0.0273 (0.0122–0.0553)	0.0215 (0.0119–0.0339)	0.0918 (0.0680–0.1042)	**	**	**
	IL-6	0.0270 (0.0000–0.049)	1.418 (0.1269–4.8495)	1.1344 (0.1020–2.7832)	16.3018 (5.1485–37.0610)	*	*	*
	IFNγ	0.0000 (0.0000–0.0000)	0.0666 (0.0000–0.2207)	0.0150 (0.0000–0.0748)	0.1793 (0.0664–0.3274)	**		**
	TNFa	0.0684 (0.000–0.1614)	0.7811 (0.2561–2.0464)	0.2644 (0.2373–0.5366)	1.6897 (0.9215–2.6344)	**		**
	GM-CSF	0.1779 (0.0000–0.5163)	0.6738 (0.0000–1.2190)	0.5376 (0.0000–0.9983)	1.8187 (0.6250–2.9474)	**	*	**
	G-CSF	0.1111 (0.0000–0.4109)	81.6 † (19.2325–115.1702)	8.7264 (3.864253–22.4210)	175.9966 (102.2350–224.3600)	**	*	**
	M-CSF	0.0775 (0.0492–0.1206)	0.3669 (0.2111–0.6281)	0.4481 (0.1236–0.8643)	14.6826 (2.4079–40.4880)	*	*	*
	KC	2.3430 (1.7789–3.2617)	19.8096 (2.0290–47.4260)	5.7795 (2.2214–12.5455)	70.1897 (37.6055–135.1504)	**	*	**
	MIP-2	1.0161 (0.3052–1.7494)	223.4 † (154.0767–271.0803)	230.60† (219.8402–241.4560)	357.2018 (246.9371–480.9945)	**	*	
	IL-12(p70)	0.0702 (0.0169–0.1461)	0.1498 (0.0987–0.2221)	0.1746 (0.0593–0.1578)	0.3607 (0.0000–0.6981)	*		
	IL-12(p40)	0.0086 (0.0000–0.0305)	0.0160 (0.0000–0.0392)	0.01974 (0.0000–0.0765)	0.0736 (0.0000–0.2041)			
	Rantes	0.0190 (0.0000–0.0335)	0.4183 † (0.2961–0.6175)	0.4276 † (0.2293–0.7087)	0.3085 (0.1040–0.4368)	*		
	IL-15	1.8561 (0.7573–3.7306)	0.5533 † (0.0000–1.5679)	0.7991 (0.0496–1.1612)	0.9902 (0.5022–1.3107)			
	IL–10	0.1976 (0.0878–0.3272)	0.2875 (0.1620–0.3606)	0.2817 (0.1698–0.4221)	0.3899 (0.1375–0.5556)			
Growth factor	VEGF	0.0621 (0.0425–0.0883)	1.1501 (0.2439–2.8221)	0.2536 (0.0873–0.6286)	3.1011 (1.6067–4.7437)	**	*	**
Remaining Th1 and Th2 cytokines	IL-2	0.5800 (0.3685–0.7409)	0.3426 (0.1942–0.5813)	0.4792 (0.3380–0.7139)	0.4275 (0.2350–0.6595)			
	IL-9	3.3111 (2.3307–4.6925)	2.5649 (1.7161–4.4603)	2.8168 (1.7129–4.9587)	2.6974 (1.6700–3.9570)			
	IL-13	0.0000 (0.0000–0.0000)	0.0794 (0.0000–0.2386)	0.0327 (0.0000–0.1635)	0.1067 (0.0000–0.2262)			
Other cytokines (tissue injury, infection, allergic diseases)	IL-5	0.0033 (0.0000–0.0131)	0.0142 (0.0000–0.0195)	0.0173 (0.0139–0.0203)	0.2271 (0.1089–0.5159)	**	**	**
	LIF	0.0241 (0.0072–0.0560)	0.2109 (0.0948–0.3703)	0.0890 (0.0356–0.1318)	3.9190 (2.0239–7.280)	**	**	**
	LIX	0.0086 (0.0000–0.0431)	0.0000 (0.0000–0.0000)	0.000 (0.0000–0.0000)	1.8256 (0.0690–3.9411)	*	*	*
	MCP-1	0.3565 (0.2727–0.5333)	3.2274 (1.2843–4.9421)	1.5886 (0.9464–1.8880)	12.1896 (6.0745–21.0912)	**	**	**
	MIP-1a	0.6512 (0.4869–0.8522)	5.2930 (0.6879–12.8468)	2.9084 (0.5998–6.7366)	37.4366 (12.7200–85.4010)	**	*	*
	MIP-1b	0.0000 (0.0000–0.0000)	2.058 (0.4973–5.3016)	1.3585 (0.6601–3.5958)	13.7665 (6.1825–25.9812)	**	**	**
	MIG	3.3655 (1.4177–5.2275)	8.3413 (2.6754–17.4490)	11.3525 (3.9570–34.2732)	5.4001 (3.3130–8.5667)			
Other cytokines (immune cell development, virus infection, aging)	IP-10	0.0963 (0.0529–0.1781)	0.8319 (0.0987–2.0276)	0.4538 (0.15725–0.7421)	1.7451 (0.7332–3.9248)	*		
	Eotaxin	0.6111 (0.2139–1.2606)	3.5192 † (0.9022–5.4013)	1.9032 (1.1310–2.8953)	4.3125 (1.0695–7.3215)	**		
	IL-3	0.0015 (0.0000–0.0075)	0.0681 (0.0183–0.1806)	0.1669 (0.0475–0.3370)	0.1577 (0.0395–0.2731)	*		
	IL-7	0.1743 (0.0000–0.7500)	0.1810 (0.0817–0.2839)	0.0696 (0.0057–0.2083)	0.2900 (0.1815–0.3531)	*		

Note: Conc., mean concentration (pg/mg) and range (minimum–maximum); †, *p* < 0.05 when compared to mock-infected group; ** *p* < 0.01; * *p* < 0.05 when USA300-C2406 compared with mock-infected group and the other 2 MRSA strains.

## Data Availability

The complete genome sequence data have been deposited at GenBank under the accession numbers: USA300-C2406 (PRJNA345240; CP019590.1), USA400-CMRSA7 (PRJNA362898), and M92 (PRJNA319679; CP015447.1).

## Supplementary Materials

The following are available online at https://www.mdpi.com/2076-2607/9/2/287/s1, Table S1: List of transcription levels for host genes related to the antibacterial response, for mice infected with USA300, USA400, and M92 MRSA strains (day 4); Table S2: Cytokine and chemokine profiles for mice on day 7 post-infection with M92, USA400, and USA300 MRSA strains; Table S3: Cytokine and chemokine profiles for mice on day 15 post-infection with M92, USA400, and USA300 MRSA strains.
