# Supplementary material for: A Murine Skin Infection Model Capable of Differentiating the Dermatopathology of Community-Associated MRSA Strain USA300 from Other MRSA Strains
Source: Microorganisms. 2021 Jan 30;9(2):287. doi: 10.3390/microorganisms9020287 (PMC7912111; doi:10.3390/microorganisms9020287)
Supplement: Supplementary file 1 [file microorganisms-09-00287-s001.zip › microorganisms-1066290-supplementary.pdf]

Supplementary Table 1. List of transcription levels for host genes related to the antibacterial response, for mice infected with USA300, USA400 and M92 MRSA strains (Day 4).

	**Comparison with Saline control**			**Comparison between strains**		
	**USA300-C2406 vs Saline** (fold regulation)	**USA400-CMRSA7 vs Saline** (fold regulation)	**M92 vs Saline** (fold regulation)	**USA300-C2406 vs USA400-CMRSA7** (fold regulation)	**USA300-C2406 vs M92** (fold regulation)	**USA4000-CMRSA7 vs M92** (fold regulation)
**Toll-Like Receptor (TLR) Signaling**						
Akt1	-	5.17	2.39	-4.49	-2.07	2.17
Casp8	3.13 **	2.78	3.26 *	-	-	-
Cd14	53.3 *	50.71	74.14	-	-	-
Fadd	-	-	-	-	-2.07	-
Irak1	-	-	-	-	-	-
Irak3	-	3.12	2.61 *	-2.43	-2.03 *	-
Irf5	-	2.22	-	-3.38	-	2.28
Irf7	5.16 *	6.46	8.2	-	-	-
Lbp	12.32 **	3.72 *	5.44 *	3.31 *	2.26 *	-
Ly96	3.42	2.47	3.96	-	-	-
Map3k7	-	-	-	-	-	-
Myd88	-	2.53	2.81	-	-	-
Pik3ca	-	-	-	-	-	-
Pstpip1	5.82 **	4.99	6.23	-	-	-
Rac1	-	-	-	-	-	-
Ripk1	-	-	-	-	-	-
Ticam1	-	-	-	-	-	-
Ticam2	4.63	6.04 ***	4.17	-	-	-
Tirap	-	-	-	-	-	-
Tlr1	22.63 **	14.37	24.33	-	-	-
Tlr2	6.75 *	7.49	9.56	-	-	-
Tlr4	7.46 *	5.78	4.88	-	-	-
Tlr5	-	-	-	-	-	-
Tlr6	4.17 *	11.43	3.83	-2.74	-	2.98
Tlr9	2.07	6.75	5.29	-3.26	-2.56	-
Tollip	-2.3 *	-	-	-2.69 *	-	-
Traf6	-	-	-	-	-	-
**NOD-Like Receptor (NLR) Signaling**						
Birc3	2.52 **	4.71	3.89 *	-	-	-
Card6	2.65 *	5.33	3.92	-2.01	-	-
Card9	3.83	5.35	4.68	-	-	-
Casp1	2.04	3.03	4.21	-	-2.06	-
Casp8	3.13 **	2.78	3.26 *	-	-	-
Hsp90aa1	3.27	2.54	2.26	-	-	-
Mefv	9.11 *	14.07	7	-	-	2.01
Naip1	-	5.17	2.39	-3.81	-	2.17
Nlrc4	2.71	5.17	3.27	-	-	-
Nlrp1a	-	7.54	2.25	-6.41	-	3.36
Nlrp3	39	48.98	48.21	-	-	-
Nod1	-	-	-	-	-	-

Nod2	-	3.11	-	-2.21	-	-
Pycard	-	-	-	-	-2.92	-
Ripk2	-	-	-	-	-2.32 **	-
Sugt1	-	-	-	-	-	-
Tnf	23.03 *	19.44	20.61	-	-	-
Xiap	-	-	2.2	-	-	-
**Other Bacterial Pattern Recognition Receptors (PRRs)**						
Apcs	6.36	5.17	5.17	-	2.67	2.17
Crp	4.63	5.17	5.17	-	-	2.17
Dmbt1	-	5.17	5.17	-4.49	-	2.17
Zbp1	7.04 **	5.16	5.16	-	-	-2.48
**Signaling Downstream of Antibacterial Responses**						
Chuk	-	-	-	-	-	-
Ikbkb	-	-	-	-	-	-
Jun	-	-	-	-	-	2.27 *
Map2k1	-	-	-	-	-	-
Map2k3	-3.55 ***	-	-2.68	-2.08 *	-	-
Map2k4	-	-	-	-	-	-
Mapk1	-	-	-	-	-	-
Mapk14	-	-	-	-	-	-
Mapk3	-	-	-	-2.13 *	-	-
Mapk8	-	-	-	-	-	-
Nfkb1	-	-	-	-	-	-
Nfkbia	-	4.09	2.72 *	-2.12	-	-
Rela	-	-	-	-	-	-
Tnfrsf1a	-	-	-	-	-	-
**Apoptosis**						
Akt1	-	5.17	2.39	-4.49	-2.07	2.17
Birc3	2.52 **	4.71	3.89 *	-	-	-
Card6	2.65 *	5.33	3.92	-2.01	-	-
Card9	3.83	5.35	4.68	-	-	-
Casp1	2.04	3.03	4.21	-	-2.06	-
Casp8	3.13 **	2.78	3.26 *	-	-	-
Cd14	53.3 *	50.71	74.14	-	-	-
Fadd	-	-	-	-	-2.07	-
Ifnb1	-	5.17	2.39	-4.49	-2.07	2.17
Ikbkb	-	-	-	-	-	-
Il12a	-	-	-	-3.2	-	-
Il12b	-	6.72	3.08	-5.83	-2.68	2.18
Il1b	420.87 *	553.19	697.25	-	-	-
Il6	3.32	5.17	6.81	-	-2.05	-
Irak1	-	-	-	-	-	-
Jun	-	-	-	-	-	2.27 *
Map3k7	-	-	-	-	-	-
Mapk1	-	-	-	-	-	-
Mapk8	-	-	-	-	-	-
Mpo	-	2.27	-	-	-	-
Nfkb1	-	-	-	-	-	-
Nfkbia	-	4.09	2.72 *	-2.12	-	-
Pik3ca	-	-	-	-	-	-

Pycard	-	-	-	-	-2.92	-
Rac1	-	-	-	-	-	-
Ripk1	-	-	-	-	-	-
Ripk2	-	-	-	-	-2.32 **	-
Tnf	23.03 *	19.44	20.61	-	-	-
Tnfrsf1a	-	-	-	-	-	-
Traf6	-	-	-	-	-	-
**Inflammatory Response**						
Akt1	-	5.17	2.39	-4.49	-2.07	2.17
Apcs	6.36	5.17	2.39	-	2.67	2.17
Ccl3	494.23 *	512.99	251.09	-	-	2.04
Ccl5	7.88 *	13.59	34.57	-	-4.39	-2.54
Cd14	53.3 *	50.71	74.14	-	-	-
Crp	4.63	5.17	2.39	-	-	2.17
Cxcl1	40.56 ***	18.23	15.9	2.23	2.55	-
Cxcl3	284.86	389.05	181.64	-	-2.07	2.14
Il1b	420.87 *	553.19	697.25	-	-	-
Il6	3.32	5.17	6.81	-	-2.05	-
Lbp	12.32 **	3.72 *	5.44 *	3.31 *	2.26 *	-
Ly96	3.42	2.47	3.96	-	-	-
Lyz2	6.15 ***	6	9.71	-	-	-
Mefv	9.11 *	14.07	7	-	-	2.01
Myd88	-	2.53	2.81	-	-	-
Nfkb1	-	-	-	-	-	-
Nlrc4	2.71	5.17	3.27	-	-	-
Nlrp3	39	48.98	48.21	-	-	-
Nod1	-	-	-	-	-	-
Rac1	-	-	-	-	-	-
Rela	-	-	-	-	-	-
Ripk2	-	-	-	-	-2.32 **	-
Slc11a1	15.39	18.89	17.15	-	-	-
Ticam2	4.63	6.04 ***	4.17	-	-	-
Tirap	-	-	-	-	-	-
Tlr1	22.63 **	14.37	24.33	-	-	-
Tlr2	6.75 *	7.49	9.56	-	-	-
Tlr4	7.46 *	5.78	4.88	-	-	-
Tlr5	-	-	-	-	-	-
Tlr6	4.17 *	11.43	3.83	-2.74	-	2.98
Tlr9	2.07	6.75	5.29	-3.26	-2.56	-
Tnf	23.03 *	19.44	20.61	-	-	-
Tnfrsf1a	-	-	-	-	-	-
Tollip	-2.3 *	-	-	-2.69 *	-	-
**Cytokines & Chemokines**						
Ccl3	494.23 *	512.99	251.09	-	-	2.04
Ccl4	147.75 *	244.17	101.82	-	-	2.4
Ccl5	7.88 *	13.59	34.57	-	-4.39	-2.54
Cxcl1	40.56 ***	18.23	15.9	2.23	2.55	-
Cxcl3	284.86	389.05	181.64	-	-2.07	2.14
Ifna9	-	5.17	2.39	-4.49	-2.07	2.17
Ifnb1	-	5.17	2.39	-4.49	-2.07	2.17
Il12a	-	-	-	-3.2	-	-

Il12b	-	6.72	3.08	-5.83	-2.68	2.18
Il18	-2.69 *	-	-	-3.22 *	-3.39 *	-
Il1b	420.87 *	553.19	697.25	-	-	-
Il6	3.32	5.17	6.81	-	-2.05	-
**Antimicrobial Peptides**						
Bpi	-	5.17	2.39	-4.49	-2.07	2.17
Camp	2.77	6.63	2.39	-2.4	-	2.78
Ctsg	-	5.17	2.39	-4.05	-	2.17
Lcn2	178.32 **	115.53	71.33 *	-	2.5	-
Ltf	2.98	10.74	4.93	-3.61	-	2.18
Lyz2	6.15 ***	6	9.71	-	-	-
Mpo	-	2.27	-	-	-	-
Prtn3	-	5.17	2.39	-3.79	-	2.17
Slpi	218.77 *	59.32	86.65	3.69	2.52	-

Note: Tested using Qiagen mouse antibacterial response PCR array. Targets are listed for each host response and some may be present in more than one group. -, transcriptional fold change was less than 2; *$p<0.05$; **$p<0.01$; ***, $p<0.001$. A negative fold change is highlighted grey.

Supplementary Table 2. Cytokine and Chemokine profiles for mice on days 7 post infection with M92, USA400 and USA300 MRSA strains.

Role	Cytokine/ Chemokine	Saline conc.	M92 conc.	USA400-CMRSA7 conc.	USA300-C2406 conc.	USA300-C2406 vs. Saline	USA300-C2406 vs. M92	USA300-C2406 vs. USA400-CMRSA7
Protection	IL-17A	0.0064<br>(0.0000-0.0065)	5.8311<br>(2.7381-9.6017)	2.6865<br>(0.3306-7.4144)	6.8417<br>(1.1541-22.2026)			
	IL-1a	27.8669<br>(16.5961-37.7149)	23.9215<br>(10.6024-33.7854)	16.6723<br>(9.6481-30.9802)	24.974<br>(14.5240-35.9588)			
	IL-1b	0.3361<br>(0.1769-0.5097)	2.2705<br>(0.4712-3.6375)	5.4559<br>(0.7044-10.4625)	3.4462<br>(2.6744-4.0097)			
Severity	IL-4	0.0076<br>(0.0025-0.0133)	0.0187<br>(0.0094-0.0275)	0.026<br>(0.0075-0.0406)	0.0434<br>(0.0218-0.0790)	*		
	IL-6	0.006<br>(0.0000-0.0115)	0.3748<br>(0.0204-1.3718)	0.9438<br>(0.0153-2.0750)	2.473<br>(0.3275-3.4872)	*	*	
	IFNγ	0.000<br>(0.0000-0.0000)	0.0806<br>(0.0000-0.1983)	0.0888<br>(0.0000-0.2734)	0.082<br>(0.0128-0.1413)			
	TNFa	0.0129<br>(0.0000-0.0327)	0.3213<br>(0.1059-0.5318)	0.5811<br>(0.1456-0.9594)	0.7073<br>(0.4138-1.0201)			
	GM-CSF	0.1813<br>(0.0000-0.4786)	0.6594<br>(0.1814-0.9258)	1.7784<br>(0.3983-5.2089)	1.1752<br>(0.9162-1.5529)			
	G-CSF	0.063<br>(0.0000-0.2607)	20.8058<br>(7.8732-50.6940)	16.1263<br>(1.9153-29.0040)	101.1745<br>(51.4449-156.3516)	**	*	*
	M-CSF	0.0504<br>(0.0337-0.0848)	0.1659<br>(0.0596-0.2600)	3.0254<br>(0.5272-11.3227)	6.2339<br>(1.4453-13.5583)			
	KC	1.7457<br>(0.6750-3.0163)	16.1875<br>(10.8424-25.7155)	22.6019<br>(5.2889-34.9305)	34.5549<br>(24.9047-39.7983)	**		
	MIP-2	0.5972<br>(0.4312-0.8887)	242.2743<br>(102.5125-382.2458)	363.2926**<br>(48.8692-773.4953)	304.0403<br>(180.8457-367.8683)	*		
	IL-12(p70)	0.0503<br>(0.0178-0.0613)	0.1486<br>(0.0680-0.3024)	0.2452<br>(0.0517-0.5429)	0.2442<br>(0.1419-0.3256)			
	IL-12(p40)	0.0065<br>(0.0000-0.0324)	0.0693<br>(0.0088-0.2145)	0.0801<br>(0.0000-0.1464)	0.0342<br>(0.0141-0.0713)			
	Rantes	0.0188<br>(0.0000-0.0373)	1.4881*<br>(0.4954-1.9930)	1.2797<br>(0.4053-2.1539)	0.3402<br>(0.2881-0.5208)			
	IL-15	0.7654<br>(0.4542-0.9879)	0.5073<br>(0.2194-0.8512)	1.1964<br>(0.0000-2.1386)	0.7485<br>(0.5604-1.1173)			
	IL-10	0.1322<br>(0.0635-0.2418)	0.1755<br>(0.0726-0.3122)	0.304<br>(0.1719-0.4953)	0.3224<br>(0.1984-0.4373)			
Growth Factor	VEGF	0.0939<br>(0.0504-0.1242)	0.8789<br>(0.0840-1.4280)	0.7044<br>(0.3731-1.0589)	1.5284<br>(1.0060-2.4036)			
Remaining Th1 and Th2 cytokines	IL-2	0.3695<br>(0.1740-0.6000)	0.2807<br>(0.2042-0.4673)	0.7114<br>(0.3525-2.0768)	0.3137<br>(0.2695-0.5203)			
	IL-9	2.8583<br>(1.2723-4.3150)	2.0577<br>(1.2930-2.8546)	5.4341<br>(1.9044-14.9804)	2.2019<br>(1.1574-2.6652)			
	IL-13	0.0019<br>(0.0000~0.0094)	0.1588*<br>(0.0000-0.3500)	0.0258<br>(0.0000-0.1291)	0.0945<br>(0.0682-0.1356)			
Other cytokines (tissue injury, infection, allergic diseases)	IL-5	0.0029<br>(0.0000-0.0145)	0.0151<br>(0.0026-0.0176)	0.0278<br>(0.0033-0.0625)	0.0603<br>(0.0188-0.1244)	*		
	LIF	0.0247<br>(0.0194-0.0333)	0.1672<br>(0.0888-0.2542)	0.2240<br>(0.0822-0.4214)	1.4260<br>(0.9997-2.0134)	**	**	**
	LIX	0.0141<br>(0.0000-0.0707)	0.2613<br>(0.0000-0.8485)	1.7353<br>(0.0000-3.7464)	0.9390<br>(0.2325-3.1900)			
	MCP-1	0.1763	6.5930**	6.2802**	5.7833	**		

		(0.0972-0.2994)	(3.7755-10.3710)	(2.4292-10.9643)	(4.5943-7.4064)		
	MIP-1a	0.6297 (0.3223-0.8892)	6.1064 (2.2334-8.6202)	10.1674 (2.6456-14.9219)	19.877 (10.5216-42.7847)	*	
	MIP-1b	0.1724 (0.0000-0.4046)	2.5765 (1.2222-4.7883)	4.0204 (1.1225-6.4906)	6.7130 (4.3510-13.0127)	**	
	MIG	2.7518 (1.1075-4.3453)	30.4542* (6.5758-49.6708)	44.9457** (26.6448-59.7023)	7.3863 (2.0267-9.3277)		**
Other cytokines (immune cell development, virus infection, aging)	IP-10	0.058 (0.0283-0.0879)	1.1581 (0.2423-2.9123)	1.7927* (0.5939-3.8250)	0.6901 (0.5343-0.8172)		
	Eotaxin	0.4300 (0.2158-0.7535)	5.9608* (3.3142-9.7373)	4.7119 (1.2508-6.6821)	5.7658* (2.1140-9.3413)		
	IL-3	0.0092 (0.0000-0.0458)	0.0731 (0.0496-0.1003)	0.1553 (0.0854-0.2589)	0.1225 (0.0426-0.2510)		
	IL-7	0.1688 (0.0000-0.3013)	0.1278 (0.0000-0.2479)	0.4066 (0.1032-1.1554)	0.2674 (0.1374-0.5000)		

Note: Con., mean concentration (pg/mg) and range (minimal-maximum); P value: ***P*<0.01; **P* <0.05; vs. saline: compared with saline control group; vs. USA400: compared with USA400-CMRSA-7 group; vs. M92: compared with M92 group.

Supplementary Table 3. Cytokine and Chemokine profiles for mice on days 15 post infection with M92, USA400 and USA300 MRSA strains.

					USA300-C2406			
**Role**	**Cytokine/ Chemokine**	**Saline conc.**	**M92 conc.**	**USA400-CMRSA7 conc.**	**USA300-C2406 conc.**	**vs. Saline**	**vs. M92**	**vs. USA400-CMRSA7**
	IL-17A	0.0090 (0.0029-0.0212)	1.0281 (0.0000-3.3931)	0.1857 (0.0107-0.5135)	0.0735 (0.0045-0.2346)			
Protection	IL-1a	29.4422 (22.6123-39.1998)	27.7803 (20.5775-34.5938)	22.9545 (8.4360-33.7729)	13.6385 (6.0992-29.4676)			
	IL-1b	0.3546 (0.2725-0.5471)	2.5540 (0.2000-7.6919)	0.8182 (0.5510-1.2965)	0.3609 (0.2563-0.1788)			
	IL-4	0.0113 (0.0046-0.0192)	0.0143 (0.0061-0.0144)	0.0170 (0.0076-0.0263)	0.0108 (0.0100-0.0115)			
	IL-6	0.0152 (0.0087-0.0216)	0.0896 (0.0184-0.1975)	1.4700 (0.0607-5.5447)	0.1444 (0.0307-0.4224)			
	IFNγ	0.0000 (0.0000-0.0000)	0.0666 (0.0000-0.2663)	0.0332 (0.0000-0.1329)	0.0000 (0.0000-0.0000)			
	TNFa	0.0421 (0.0233-0.0580)	0.3565 (0.0486-0.7644)	0.2022 (0.1102-0.2792)	0.0928 (0.0716-0.1364)			
	GM-CSF	0.2183 (0.0000-0.8731)	0.5481 (0.0688-1.2781)	0.5328 (0.0000-0.9740)	0.4241 (0.1738-0.6341)			
	G-CSF	0.0205 (0.0000-0.0231)	0.6450 (0.0000-2.2750)	1.1249 (0.0362-2.9980)	1.4913 (0.0637-5.0381)			
	M-CSF	0.0395 (0.0250-0.0708)	0.1489* (0.0461-0.2163)	0.0981 (0.0417-0.1553)	0.1041 (0.0626-0.1506)			
Severity	KC	1.1661 (0.9109-1.6779)	6.7590 (1.0127-18.1375)	5.7285 (1.7078-11.9637)	1.6732 (0.6454-2.8646)			
	MIP-2	0.4294 (0.3077-0.5814)	4.9329 (0.8691-14.2294)	8.9785 (0.5083-22.1577)	1.2638 (0.3497-2.2843)			
	IL-12(p70)	0.044 (0.0356-0.0525)	0.1478 (0.0598-0.2756)	0.0870 (0.0486-0.1444)	0.1031 (0.0565-0.1548)			
	IL-12(p40)	0.0073 (0.0000-0.0125)	0.0198 (0.0000-0.0430)	0.0098 (0.0000-0.0390)	0.0005 (0.0000-0.0023)			
	Rantes	0.0101 (0.0000-0.0404)	13.1815 (0.0505-45.1938)	1.6185 (0.3200-3.0024)	0.4126 (0.0887-1.1489)			
	IL-15	0.8604 (0.6116-1.4154)	1.6896 (0.5209-2.0494)	1.0279 (0.4502-1.9673)	0.4760 (0.1556-0.8995)		*	
	IL-10	0.1416 (0.0558-0.2984)	0.2258 (0.0693-0.3869)	0.0738 (0.0000-0.2097)	0.0894 (0.0237-0.1571)			
Growth Factor	VEGF	0.0481 (0.0216-0.0696)	0.6309 (0.0761-1.7319)	0.3147 (0.0510-0.4547)	0.2878 (0.0280-0.8604)			
	IL-2	0.3407 (0.2680-0.5141)	0.5277 (0.3391-0.5763)	0.4925 (0.2278-0.9776)	0.2682 (0.0497-0.6425)			
Remaining Th1 and Th2 cytokines	IL-9	2.149 (1.6946-3.0917)	4.6561 (2.2207-6.0481)	3.505 (2.2521-5.3816)	1.9686 (1.0104-3.6475)		*	
	IL-13	0.000 (0.0000-0.0000)	0.0075 (0.0000-0.0300)	0.0814 (0.0000-0.2981)	0.0000 (0.0000-0.0000)			
	IL-5	0.0124 (0.0000-0.0284)	0.0147 (0.0050-0.0245)	0.0403 (0.0212-0.0796)	0.0227 (0.0186-0.02448)			
Other cytokines (tissue injury, infection, allergic diseases)	LIF	0.0278 (0.0104-0.0587)	0.2737 (0.0230-0.6988)	0.1521 (0.0795-0.1248)	0.1222 (0.1057-0.1329)			
	LIX	0.0000 (0.0000-0.0000)	0.0370 (0.0000-0.1481)	0.0000 (0.0000-0.0000)	0.0000 (0.0000-0.0000)			
	MCP-1	0.2798 (0.2110-0.3102)	7.8279 (0.3741-25.9475)	2.3032 (1.6291-3.0486)	1.9772 (1.2228-2.3272)			

	MIP-1a	0.3600 (0.2896-0.4827)	2.4247 (0.6000-6.2544)	0.9225 (0.2205-1.4191)	0.3675 (0.1697-0.9529)	
	MIP-1b	0.0659 (0.0000-0.2637)	1.4033 (0.0000-3.8269)	0.7749 (0.3169-1.0397)	0.3043 (0.2222-0.5454)	
	MIG	2.1910 (0.9971-4.7288)	69.3482* (3.0230-132.9413)	22.5006 (2.7523-36.8708)	12.3011 (0.5971-38.7418)	*
Other cytokines (immune cell development, virus infection, aging)	IP-10	0.0493 (0.0311-0.0673)	1.3861 (0.0500-4.0469)	0.3682 (0.1093-0.5528)	0.2124 (0.0465-0.5418)	
	Eotaxin	0.2368 (0.2038-0.2827)	2.5170 (0.3016-5.7044)	0.7363 (0.6500-0.9188)	1.1944 (0.6307-3.2329)	
	IL-3	0.0033 (0.0000-0.0032)	0.0506 (0.0250-0.0975)	0.0314 (0.0000-0.0796)	0.0531 (0.0288-0.1271)	
	IL-7	0.0493 (0.0000-0.0981)	0.2192 (0.0000-0.4519)	0.2090 (0.1067-0.2533)	0.1509 (0.0892-0.1681)	

Note: Con., mean concentration (pg/mg) and range (minimal-maximum); P value: **P<0.01; *P <0.05; vs. saline: compared with saline control group; vs. USA400: compared with USA400-CMRSA-7 group; vs. M92: compared with M92 group.
